# Deep-Learning and Device-Assisted Enteroscopy: Automatic Panendoscopic Detection of Ulcers and Erosions

**DOI:** 10.3390/medicina59010172

**Published:** 2023-01-15

**Authors:** Miguel Martins, Miguel Mascarenhas, João Afonso, Tiago Ribeiro, Pedro Cardoso, Francisco Mendes, Hélder Cardoso, Patrícia Andrade, João Ferreira, Guilherme Macedo

**Affiliations:** 1 Precision Medicine Unit, Department of Gastroenterology, São João University Hospital, Alameda Professor Hernâni Monteiro, 4200-427 Porto, Portugal; 2 WGO Gastroenterology and Hepatology Training Center, 4200-427 Porto, Portugal; 3 Faculty of Medicine of University of Porto, Alameda Professor Hernâni Monteiro, 4200-427 Porto, Portugal; 4 Department of Mechanical Engineering, Faculty of Engineering of the University of Porto, Rua Dr. Roberto Frias, 4200-465 Porto, Portugal; 5 DigestAID—Digestive Artificial Intelligence Development, Rua Alfredo Allen, 455/461, 4200-135 Porto, Portugal

**Keywords:** device-assisted enteroscopy, ulcerative lesions, artificial intelligence, convolutional neural networks, deep learning

## Abstract

*Background and Objectives*: Device-assisted enteroscopy (DAE) has a significant role in approaching enteric lesions. Endoscopic observation of ulcers or erosions is frequent and can be associated with many nosological entities, namely Crohn’s disease. Although the application of artificial intelligence (AI) is growing exponentially in various imaged-based gastroenterology procedures, there is still a lack of evidence of the AI technical feasibility and clinical applicability of DAE. This study aimed to develop and test a multi-brand convolutional neural network (CNN)-based algorithm for automatically detecting ulcers and erosions in DAE. *Materials and Methods*: A unicentric retrospective study was conducted for the development of a CNN, based on a total of 250 DAE exams. A total of 6772 images were used, of which 678 were considered ulcers or erosions after double-validation. Data were divided into a training and a validation set, the latter being used for the performance assessment of the model. Our primary outcome measures were sensitivity, specificity, accuracy, positive predictive value (PPV), negative predictive value (NPV), and an area under the curve precision–recall curve (AUC-PR). *Results*: Sensitivity, specificity, PPV, and NPV were respectively 88.5%, 99.7%, 96.4%, and 98.9%. The algorithm’s accuracy was 98.7%. The AUC-PR was 1.00. The CNN processed 293.6 frames per second, enabling AI live application in a real-life clinical setting in DAE. *Conclusion*: To the best of our knowledge, this is the first study regarding the automatic multi-brand panendoscopic detection of ulcers and erosions throughout the digestive tract during DAE, overcoming a relevant interoperability challenge. Our results highlight that using a CNN to detect this type of lesion is associated with high overall accuracy. The development of binary CNN for automatically detecting clinically relevant endoscopic findings and assessing endoscopic inflammatory activity are relevant steps toward AI application in digestive endoscopy, particularly for panendoscopic evaluation.

## 1. Introduction

Ulcers and erosions are the most prevalent lesions of the small bowel [1,2]. Indeed, ulcerative and erosive lesions of the small intestine have been easier to detect since the introduction of capsule endoscopy (CE) in the early 2000s [3]. The etiology of these lesions is vast and can be associated with non-steroid anti-inflammatory drugs, as well as many systemic diseases, like Crohn’s disease, refractory celiac disease, neoplasms, and infections [4].

While the introduction of CE exponentially increased the capacity of mucosa evaluation and detection of bowel lesions, its purely diagnostic nature demanded developing therapeutic deep enteroscopy procedures. Device-assisted enteroscopy (DAE), which comprehends single- and double-balloon enteroscopy, plus motorized spiral enteroscopy, enabled gastroenterologists to sample tissue and treat lesions [5]. DAE has a significant role in the approach of small intestinal lesions, mainly after a positive CE [5,6]. Although the clinical setting and characterization of ulcerative lesions in CE may suggest probable etiologies, no distinctive macroscopic aspect can be used to make a definitive diagnosis. Moreover, revising CE videos is monotonous and prone to errors and missing lesions. DAE might be crucial in these cases by contributing to a correct histological final diagnosis (i.e., excluding infectious enteropathy or the presence of malignant cells).

Artificial intelligence (AI) application in gastroenterology is growing exponentially in many areas, such as gastrointestinal upper and lower endoscopy and hepatology [7,8,9,10]. Implementing AI algorithms for CE evaluation has also shown promising improvements, making the assessment of intestinal mucosa less time-consuming [4,11]. Despite significant advances in efficiency and identification of lesions by applying deep-learning technologies in CE, there is still a lack of evidence of AI pertinence during DAE. Although AI application during DAE has been studied to detect vascular and protruding enteric lesions automatically, no proof-of-concept studies in ulcerative lesions have been conducted [12,13].

This study aimed to develop and test a CNN-based algorithm for automatically detecting ulcers and erosions in DAE.

## 2. Materials and Methods

### 2.1. Study Design and Data Collection

Patients consecutively submitted to DAE between January 2020 and July 2022, regardless of intubation direction, at a single tertiary center (Centro Hospitalar Universitário São João, in Porto, Portugal) were included (n = 250). In that period, DAE was performed by two experienced endoscopists using the double-balloon enteroscopy system Fujifilm EN-580T (n = 152) and the single-balloon enteroscopy system Olympus EVIS EXERA II SIF-Q180 (n = 98). Recorded videos from all these procedures were collected retrospectively. Images from the esophagus, stomach, small intestine, and colon were recovered. Each video was segmented in still frames using dedicated video software (VLC media player, Paris, France).

Each extracted frame was then evaluated for the presence of ulcers or erosions, defined as mucosal breaks with white or yellow bases, surrounded by reddish or pink mucosa [14]. Images were divided into two groups, one with normal mucosa and the other with ulcerative lesions. The final classification of each frame required a consensus between three experienced gastroenterologists. When a common agreement was not possible, the frame was excluded. A total of 6772 images were used, of which 633 contained ulcers or erosions.

### 2.2. Development of the CNN and Performance Analysis

**Figure 1:**
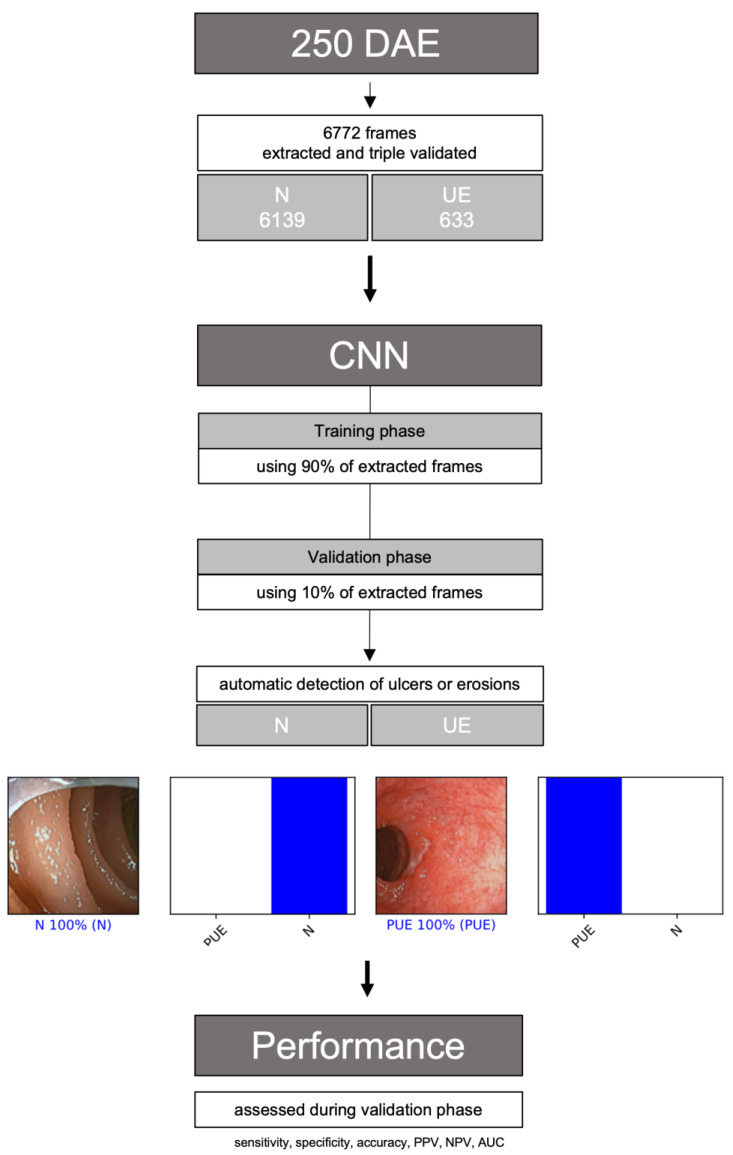
Flowchart describing the study design. N: normal mucosa; UE: ulcers or erosions; PPV: positive predictive value; NPV: negative predictive value; AUC-PC: area under the precision–recall curve.

A deep-learning convolutional neural network (CNN) was constructed to detect gastrointestinal erosions or ulcers automatically, enabling panendoscopic inflammatory assessment. From the complete data set, 90% (n = 6094) was used to develop and train the algorithm. The remaining 10% (n = 678) was used to validate CNN performance independently. Figure 1 represents a graphical flowchart of the study design.

We used the XCeption model to build the CNN. Weights between units were trained on ImageNet, a large-scale image dataset created for object software recognition. We conserved its convolutional layers to transfer its learning to our model. We removed the last fully connected layers from our own classifier of dense and dropout layers. Each of the two blocks we used had a completely connected layer first, then a dropout layer with a 0.2 drop rate. Finally, we added a dense layer whose size determined the number of categories to be classified (two: normal or ulcers/erosions). The learning rate (varying between 0.0000625 and 0.0005), batch size of 128, and the number of epochs of 20 were set by trial and error. The model was prepared using PyTorch and scikit libraries [15,16]. Standard data augmentation techniques, including image rotations and mirroring, were applied during the training stage. The computers were powered with a 2.1 GHz Intel^®^ Xeon^®^ Gold 6130 processor (Intel, Santa Clara, CA, USA) and a double NVIDIA Quadro^®^RTXTM 8000 graphic processing unit (NVIDIA Corp, Santa Clara, CA, USA).

**Figure 2:**
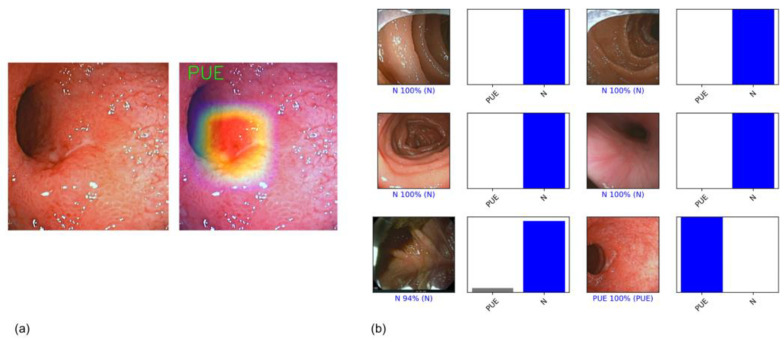
(**a**) Example of a heatmap highlighting CNN’s detection of an ulcerative; (**b**) output obtained from CNN application. Each bar represents the probability estimated by the algorithm of being considered normal and a probability of having ulceration or erosion. Each frame was classified in one of the aforementioned categories considering the one with higher probability. The corresponding evaluation provided by the three expert endoscopists, which was considered the gold standard, is written between parentheses. In the case of a correct prediction, the bar was painted blue. In the case of an incorrect one, the bar was painted red.

For each frame, the algorithm calculated the probability of being considered normal and a probability of having mucosa with ulcers or erosion. Each frame was classified into one of the categories above, selecting the one with the higher probability (Figure 2). The algorithm’s final classification of each image was compared with the corresponding evaluation provided by the two experts, the latter being considered the gold standard. Our primary outcome measures were sensitivity, specificity, accuracy, positive predictive value (PPV), negative predictive value (NPV), and area under the precision–recall curve (AUC-PR). The interpretation of a conventional receiver operating characteristic (ROC) curve can be misleading in cases of an imbalanced dataset, with a much higher proportion of normal mucosa frames (true negatives) than frames containing ulcerative lesions (true positives) [17]. This is explained by the ratio of true negatives, which can cause the ROC curve to shift to the left without necessarily implying improved diagnostic accuracy. Thus, we decided to use the precision–recall metric, since it is unaffected by true negatives.

**Figure 3:**
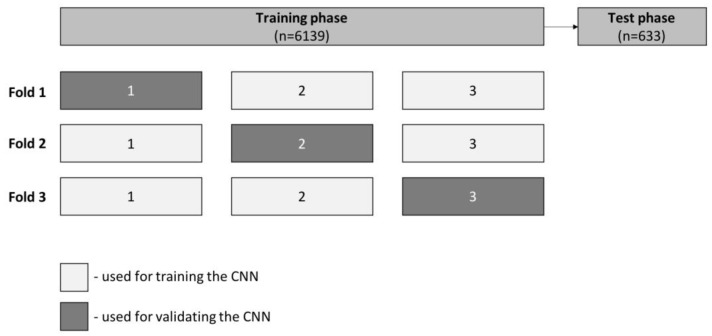
Schematic representation of three-fold cross-validation performed during the training phase. Frames were randomly divided into three equal groups from the total training set. We used two of the three created groups (66.7%) for training and one (33.3%) for model validation. We ran three runs with the aforementioned proportions but varying the groups used for training and validation. The remaining 10% of the data set was used to test CNN performance independently, during the validation phase.

At the training stage, which included 90% of the full dataset, we performed three-fold cross-validation. At this experiment, for each fold the training dataset was divided into three even-sized groups. Within each fold, two groups were used for training (66.7%) and one group for validation (33.3%). For each fold, the groups used for training and validation varied, as shown in Figure 3. Sensitivity, specificity, accuracy, PPV, NPV and AUC were calculated for each run. The remaining 10% of the dataset was used to test CNN performance independently (test phase), using model specifications associated with the best-trained model in the previous phase. We also evaluated its computational performance by calculating the algorithm processing time of all frames from the validation set. We used Sci-Kit Learn v0.22.2 to perform statistical analysis [18].

## 3. Results

### 3.1. CNN Development during Training Phase

A total of 6772 images were used, of which 633 were considered ulcers or erosions after double-validation. From the full data set, 90% (n = 6094) was used to develop and train the algorithm.

The results of the three-fold cross-validation experiment are shown in Table 1. The mean model sensitivity and specificity were 89.7% (CI 95%: 84.2–95.6%) and 99.5% (CI 95%: 99.2–99.8%). The mean CNN accuracy was 98.6% (CI 95%: 98.1–99.1%).

### 3.2. CNN Performance during Test Phase

The remaining 10% (n = 678) of data were used to validate CNN performance independently.

The distribution of the main outcomes is shown in Table 2. The sensitivity and specificity of the model were 88.5% and 99.7%, respectively. The PPV was 96.4% and the NPV was 98.9%. Overall, our algorithm accuracy was 98.7%. The AUC-PR predicting enteric ulcers and erosions was 1.00. The CNN algorithm had a reading rate of approximately 293.6 frames per second.

## 4. Discussion

This is the first study evaluating AI applicability in the automatic detection of ulcers and erosions in DAE. Our findings suggest that the automatic detection of ulcerative lesions with this algorithm can be performed effectively, as it achieved high sensitivity and specificity and may be feasible during real-time procedures. Furthermore, this model is the first to automatically detect ulcers and erosions throughout the entire digestive tract during enteroscopy, paving the way to panendoscopical AI analysis.

Our findings show that the performance of our algorithm levels was high, with sensitivity and specificity levels of 88.5% and 99.7%, respectively, and an overall diagnostic accuracy level of 98.9%. This is conjugated with a high processing frame rate capacity, with a reading rate of 294 frames per second. This fact ensures the real-time applicability of the AI model for endoscopic analysis, increasing the technology readiness level (TRL) of the developed AI algorithm. In addition, our CNN was constructed through acquired data from two different models of DAE, which may also increase the TRL of technology and its usefulness in real-life clinical practice, solving significant inter-operability challenges.

These findings are consistent with previous research on the automatic detection of ulcerative lesions using other gastroenterology procedures. Regarding CE, Aoki et al. demonstrated good diagnostic performance in automatically detecting ulcers and erosions, with a sensitivity level of 88.2%, specificity level of 90.9%, and accuracy of 90.8% [4]. Another group reported the development of a CNN for detecting mucosal breaks during CE, which can also predict their bleeding potential, with sensitivity, specificity and accuracy levels of 90.8%, 97.1% and 95.1%, respectively [19]. AI models also achieve good diagnostic performance metrics at colon capsule endoscopy (CCE), with a sensitivity level of 96.9%, specificity level of 99.9%, and accuracy of 99.6% for automatically detecting ulcers and erosions [20]. AI not only enables proficuous pandendoscopic noninvasive evaluation, but may also offer valuable guidance in device-assisted enteroscopy, providing full guidance in the endoscopic diagnostic workflow.

This study has some limitations. On the one hand, this was a unicentric study based on retrospective data, which might be associated with a selection bias. On the other hand, the number of frames used to develop and test this model was relatively small, thus compromising the external validity of our results. Although our results look promising, the risk of overfitting should not be omitted. Therefore, a final conclusion cannot be reached regarding its applicability in everyday situations. Multicentric studies are needed, with more extensive and prospective data collection, to ensure appropriate dataset variability. Moreover, it should be noted that anatomopathological diagnosis of Crohn’s disease remains challenging, since there is a higher proportion of false-negatives when biopsies are taken during enteroscopy [21]. Clinical and non-endoscopic imaging techniques remain crucial, namely when a strong suspicion exists, despite the microscopic result being negative. AI applications, namely AI guided biopsies, during device-assisted enteroscopy, may increase diagnostic yields of this technique, particularly for Crohn’s disease.

## 5. Conclusions

AI application is growing in various imaged-based gastroenterology procedures, considering that it might lead to higher quality endoscopy, better clinical decisions, and still potentially be cost-effective. This study is a continuation of our previous foundational studies regarding AI and DAE. It suggests that automatic detection of ulcers and erosions using a CNN during DAE is associated with high overall accuracy. Furthermore, implementation of this CNN during DAE might contribute to a higher detection rate of ulcerative lesions, given by real-time feedback during the procedure, which in turn may contribute to lower inter-observer variability and a lower false-negative rate.

## Figures and Tables

**Table 1:** Three-fold cross-validation, which was performed during the training phase.

	Sensitivity (%)	Specificity (%)	PPV (%)	NPV (%)	Accuracy (%)
Fold 1	88.5	99.4	93.4	98.8	98.4
Fold 2	87.9	99.7	97.1	98.8	98.6
Fold 3	93.2	99.5	94.7	99.3	98.9
Overall mean (95% CI)	89.7 (84.2–95.6)	99.5 (99.2–99.8)	95.1 (91.4–98.7)	99.0 (98.4–99.5)	98.6 (98.1–99.1)

PPV: positive predictive value; NPV: negative predictive value.

**Table 2:** Confusion matrix of the test set versus expert classification (considered the final diagnosis).

		Final Diagnosis	
		UE	N
CNN classification	UE	54	2
	N	7	615

CNN: convolutional neural network; N: normal mucosa; UE: ulcers or erosions.

## Data Availability

Not applicable.
